# Developing core indicators for identifying people at risk of delayed heart failure diagnosis

**DOI:** 10.1186/s12875-025-03024-4

**Published:** 2025-10-16

**Authors:** K. Barber, L. Bernhardt, GP McCann, I. Squire, CA Miller, C. Deaton, K. Khunti, C. A. Lawson

**Affiliations:** 1 Leicestershire Partnership NHS Trust, Leicester, UK; 2 Department of Cardiovascular Sciences, NIHR Cardiovascular Biomedical Research Centre, University of Leicester, Glenfield Hospital, Leicester, Leicestershire, England LE5 4PW UK; 3 Division of Cardiovascular Sciences, School of Medical Sciences, Faculty of Biology, Medicine and Health, Manchester Academic Health Science Centre, University of Manchester, Manchester, UK; 4 Manchester University NHS Foundation Trust, Southmoor Road, Manchester, Wythenshawe UK; 5 Wellcome Centre for Cell-Matrix Research, Division of Cell-Matrix Biology & Regenerative Medicine, School of Biology, Faculty of Biology, Medicine & Health, Manchester Academic Health Science Centre, University of Manchester, Manchester, UK; 6 Department of Public Health and Primary Care, School of Clinical Medicine, University of Cambridge, Cambridge, UK; 7 Leicester Real World Evidence Unit, Diabetes Research Centre, University of Leicester, Leicester, UK

**Keywords:** Heart failure, Diagnosis, Consensus

## Abstract

**Background:**

Heart failure (HF) is frequently diagnosed during hospital admission, often after symptoms have been present for some time. Those diagnosed in hospital typically experience higher mortality reflecting not only possible diagnostic delay but also greater illness severity at presentation. The reasons behind delayed HF diagnosis are multifaceted and complex. This study aimed to achieve consensus on a priority list of patient, clinical, and service-level factors associated with delayed HF diagnosis, and to identify indicators that could support earlier detection of undiagnosed HF in primary care.

**Methods:**

A three-round modified e-Delphi process involved patients and clinicians from primary and specialist care. Participants rated sociodemographic and clinical factors for their importance in delayed HF diagnosis and clinicians also rated service-level factors and identified indicators of undiagnosed HF. Consensus was defined as two-thirds agreement with stable opinions across rounds (McNemar *p* ≥ 0.05). Indicators of undiagnosed HF required additional ranking in the top 5 by > 50% of clinicians.

**Results:**

The first survey was completed by 18 patients (67% women, median age 61) and 27 clinicians (67% nurses/allied health professionals, 33% doctors). Consensus was achieved, comprising 15 factors and 5 indicators. Key sociodemographic factors were patients lacking HF knowledge, lack of access to GP/cardiologist appointments, symptom confusion, younger age (< 50), and learning difficulties. Clinical factors included multimorbidity, respiratory/mental health conditions, obesity, and depression. Service-level factors included poor HF knowledge, limited N-terminal pro–B-type Natriuretic Peptide (NT-proBNP) testing and echocardiogram access in primary care, and fragmented care. The top 5 indicators of undiagnosed HF included elevated NT-proBNP without referral, loop diuretic use, and overlapping cardiac and respiratory histories.

**Conclusions:**

This study identifies critical factors and indicators that can aid earlier HF diagnosis in primary care. These indicators could be embedded into electronic health record–based alerts and used to support decision-making in primary care.

**Supplementary Information:**

The online version contains supplementary material available at 10.1186/s12875-025-03024-4.

## Introduction

Heart failure (HF) often goes undiagnosed until patients are hospitalised [1], and those diagnosed in hospital typically experience higher mortality [2] and healthcare costs [3], reflecting not only possible diagnostic delay but also greater illness severity at presentation. Much of the increased cost relates to the expense of hospital-based care rather than community management. As the leading cause of preventable hospitalisations in Europe [4], early diagnosis and treatment are now key policy priorities [5]. 

HF diagnosis begins with patient-reported symptoms and recognition in primary care, but formal confirmation usually requires N-terminal pro–B-type Natriuretic Peptide (NT-proBNP) testing, echocardiography and specialist review [6, 7]. Access to echocardiography varies nationally, and waiting times for specialist assessment can be long. The average time from symptom onset to HF diagnosis can exceed two years [8], with disproportionately longer delays for women, older adults, individuals living in deprived areas, and those with multiple long-term conditions. The reasons behind delayed HF diagnosis are multifaceted and complex. System-level barriers [9], such as limited access to diagnostic tests or specialist services, clinician-level factors [10], including knowledge gaps, diagnostic uncertainty, or variable interpretation of symptoms and patient-level barriers [11, 12], including symptom recognition and reporting, all contribute to these delays. While most newly diagnosed HF patients initially present in primary care, fewer than half report symptoms, and only a quarter receive recommended diagnostic tests or specialist referrals [1]. Inappropriate and repeated testing is also prevalent [13], further prolonging diagnosis.

Improving the diagnosis and management pathway is essential for national efforts aimed at reducing hospitalisations and mortality associated with HF [14, 15]. Identifying patients at the highest risk of undiagnosed HF in primary care is critical to reducing unnecessary testing and accelerating accurate diagnosis. Through consensus methods involving HF patients and clinicians, we aimed to identify a core set of patient, clinical, and service-level risk factors for delayed HF diagnosis in primary care, as well as key indicators of undiagnosed HF.

## Methods

### Study design

Consensus study using Delphi methods.

### Participants

Participants included individuals with HF from hospital and community settings in Leicestershire in the UK, as well as from a national HF patient charity network. Patients were eligible if they:

- Were aged 18 years or older.
- Had a confirmed HF diagnosis.
- Had a self-reported HF diagnosis and were under the care of a HF specialist.
- Had access to an electronic device (e.g., phone, computer, or tablet).
- Could speak English.
- Were willing and able to provide informed consent.

No exclusions were made based on the type or severity of HF. Patients were recruited through community and hospital HF teams and a national patient-led HF charity. For those identified via the clinical teams, their HF diagnosis was verified by the clinical team as part of routine care using European Society of Cardiology criteria [6], which include a combination of signs, symptoms, and objective evidence of cardiac abnormality. Patients recruited through the national network self-reported their HF diagnosis. Exclusions were made for patients unlikely to survive hospital admission or nearing the end of life.

To ensure inclusion of participants with relevant expertise, clinicians were purposively sampled and eligible to participate if they were registered nurses or doctors (including general practitioners, cardiologists, and other medical doctors) with:

- A minimum of one year of post-registration clinical experience.
- Specific experience in HF care.

This purposive approach was appropriate for a consensus study, as it ensured that participants had the knowledge and experience necessary to provide informed input on risk factors and indicators for delayed HF diagnosis.

### Recruitment and consent

The study was conducted between March 26, 2024, and July 17, 2024. Potential patient participants were invited via personalised letters containing study details. Patients who expressed interest were provided with a digital consent form for completion. Clinicians were identified through various routes, including service managers within their community or hospital trusts, an open invitation to a national HF clinical network, and personal contacts with international HF experts. Clinicians indicated their willingness to participate through tacit consent, confirmed by email and through completion of the Delphi surveys. All participants were informed of their right to withdraw from the study at any point. However, data collected prior to withdrawal would remain part of the consensus findings.

### Sample size

Previous evidence indicates that 11–25 participants are needed to achieve consensus [16]. Therefore, the recruitment target was set to include at least 30 participants (15 clinicians and 15 patients).

### Ethical review

The study was conducted in accordance with the principles outlined in the Declaration of Helsinki. Ethics approval was granted by the Health and Social Care Research Ethics Committee A (HSC REC A) [23/NI/0156].

### Data governance

Each participant was assigned a unique study ID, and all baseline and questionnaire data were pseudo-anonymised before transfer to a secure university network managed by a designated data custodian. Access to this data was regulated under current governance protocols. Participants’ email addresses were stored separately from study data and promptly deleted following the distribution of study feedback.

### Participant characteristics

The first survey included self-reported socio-demographic information from patients (age, sex, ethnicity and social status) as well as clinical details such as HF diagnosis, comorbidities, and diagnostic history. Clinicians were asked to provide baseline data including sex, ethnicity, professional role, and healthcare experience.

### Modified e-Delphi process and statistical analysis

The Delphi method is a structured technique used to achieve consensus by collecting and analysing individual participant feedback, particularly in contexts where a variety of opinions are anticipated [17] For this study, a modified e-Delphi method [18] was employed to achieve group consensus over the course of three rounds. Items for inclusion in the electronic questionnaires were drawn from published literature [19–21] and discussions with patients and clinicians [14, 22] (Table 1).

**Table 1:** Delphi questionnaire items

Sociodemographic factors	
Being Young (< 50 years)/being Old (> 80 years)	*Health literacy & Access to services*
Being Male/being Female	- No transport
Being affluent/being deprived	- No internet
*Ethnicity*	- Limited education
- White	- Learning difficulty
- South Asian	- No English
- Black	- No heart failure knowledge
*Social care and support*	
- Living alone	
- Being a carer	

**Clinical conditions**	
Respiratory condition	Overweight
Other conditions (multimorbidity)	Depression
Mental health condition	Kidney problems
Polypharmacy	Diabetes

**Service level factors (clinicians only)**	
Lack of HF knowledge in primary care settings	Lack of HF knowledge in non-HF hospital settings
Lack of HFpEF recognition in primary care settings	Lack of HFpEF recognition in non-HF hospital settings
Lack of BNP testing in primary care settings	Lack of BNP testing in hospital settings
Lack of echocardiogram access in primary care settings	Lack of echocardiogram access in hospital settings
Lack of detailed echocardiogram reports in primary care settings	Lack of discharge provision from hospital
Lack of skills read echocardiogram in primary care settings	Fragmented care

**Indicators of undiagnosed HF (clinicians only)**	
Loop diuretic only (no cardiac or respiratory history)	Symptoms only
Loop diuretic and cardiac history (no respiratory history)	Repeat symptoms only
Loop diuretic and respiratory history (no cardiac history)	Oedema only (no cardiac/respiratory history or Loop diuretic)
Cardiac and respiratory history (no Loop diuretic)	High BNP but no referral or diagnosis
Cardiac history only (no respiratory history or Loop diuretic)	HF care plan but no diagnosis or follow-up

Initial Delphi questionnaire items drawn from published literature and discussions with patients and clinicians

For patients, a web-based survey presented a list of sociodemographic and clinical factors that might influence the diagnosis of HF, such as age, socioeconomic status and multimorbidity. For clinicians, the web-based survey was in two sections: (i) a list of sociodemographic, clinical and service level factors (e.g. access to diagnostic tests) that could impact HF diagnosis and (ii) a list of potential criteria indicating undiagnosed HF in patients presenting with symptoms suggestive of HF.

Participants received three surveys, one per month for three months, via an email link.

*Round one:* Patients and clinicians were asked to rate their agreement with two statements.

(i) “The following patient factors are important factors that might delay diagnosis of heart failure”.
(ii) “The following clinical factors are important factors that might delay diagnosis of heart failure”.
  Clinicians were also asked to evaluate two additional statements:
(iii) “The following are important service level factors that might delay diagnosis of heart failure”.
(iv) “In patients in general practice without a diagnosis of heart failure currently, the following are important indicators of undiagnosed heart failure”.

Participants provided importance ratings on Likert scales including 1 (“Strongly disagree”), 2 (“Disagree”), 3 (“Agree”), 4 (“Strongly agree”) and 5 (“Strongly agree”). Participants could also suggest additional factors if they felt important ones were missing. Items were carried forward to Round 2 if they received a mean score of greater than 3 (leaning towards ‘agree’ or ‘strongly agree’). For sociodemographic and service-level factors, where the initial items exceeded 10, only the top 10 scoring items in each category were carried forward.

Patient and clinician questionnaires can be found in the supplementary information (S1 and S2 Figures).

*Round two:* Participants were shown the average scores for each factor from Round 1 and asked to re-evaluate and re-rate their responses. To assess changes in agreement between rounds, we applied McNemar’s test, a repeated-measures chi-square test that evaluates shifts in paired proportions [23]. For this analysis, responses of agree or strongly agree were coded as “yes” (include), while undecided, disagree, and strongly disagree were coded as “no” (don’t include). A p-value ≥ 0.05 was interpreted as indicating stability, i.e., no significant change in the proportion of agreement between rounds. In addition, clinicians were asked to rank the most important indicators of undiagnosed HF.

In this round, factors were considered to achieve consensus if they met the following two criteria:

(i) At least 66% of participants (two-thirds threshold) either agreed or strongly agreed with a factor’s importance.
(ii) The factor’s importance remained stable between rounds (McNemar *P* ≥ 0.05).

For clinical indicators of undiagnosed HF, factors also had to be ranked in the top five by more than 50% of clinicians to achieve consensus, a pragmatic threshold chosen to ensure that only indicators with broad support were retained.

*Round 3:* Participants were asked to rank all the factors that achieved consensus in order of importance, from highest to lowest priority.

*Sensitivity analyses:* In Round 1, item selection and group feedback were based on mean scores. Although Likert-scale data are ordinal, the mean is commonly used in Delphi studies because it can provide greater sensitivity than the median [24], which in our dataset frequently yielded identical values across items and was therefore less informative for participant feedback. The mean was applied descriptively for selection and to guide feedback and re-rating, not for parametric statistical testing. From Round 2 onwards, consensus was defined a priori as at least two-thirds (66%) of participants selecting “agree” or “strongly agree.” To test robustness, we re-examined Round 1 data using a broader definition of consensus (two-thirds of participants selecting “undecided,” “agree,” or “strongly agree”), corresponding to the mean score ≥ 3 threshold.

To assess the potential impact of attrition across rounds, we conducted a sensitivity analysis restricted to participants who completed all three rounds (“completers analysis”). Consensus thresholds were re-applied to this subgroup to evaluate whether the pattern of retained items differed from the full sample.

#### Patient and public involvement

Patient and stakeholder input has been essential to the study aims and design. Over 45 national clinical, public health, patient and social care representatives attending a HF summit (‘25in25’) identified key challenges of HF diagnosis, including sociodemographic inequalities and comorbidity. These challenges were reiterated in two consultation groups with ten HF patients. Patients often reported experiencing symptoms leading to multiple consultations with their GP. Frequently, they were only told their diagnosis following admission to hospital.

## Results

### Participants

The initial survey involved 18 patients (67% women, median age 61 [IQR 51–65] years and 27 clinicians (67% nurses/AHPs, 33% doctors) (Table 2). Most patients were able to live independently, without requiring care (72%) and had two or more comorbidities (67%). Symptom burden was high, with 78% experiencing shortness of breath, 67% reporting fatigue, and 44% reporting ankle swelling. Most patients (61%) had been living with HF for over five years, and 72% were diagnosed during a hospital admission after a median of 7 months (range 3–12) of feeling unwell. Nearly all clinicians had over 10 years of experience post-professional registration (93%), and 52% had worked in HF care for more than 10 years. Regarding their practice settings, 11 (41%) worked in a HF community or general practice setting, 8 (30%) in a HF hospital setting, and 8 (15%) in non-HF settings which included acute or general cardiology, urgent care, general practice and research.

**Table 2:** Participant information

Patients (*n* = 18)	
Age; median years (1st and 3rd quartiles)	61 (51, 65)
Women	12 (66.7)
Men	6 (33.3)
≥ 2 comorbidities	12 (66.7)
Care requirements	
- Independent	13 (72.2)
- Requires care	5 (27.8)
Lifestyle factors	
- Never smoked	9 (50)
- Ex-smoker	9 (50)
- Current alcohol drinker	10 (55.6)
- BMI; median (1st and 3rd quartiles)	33.9 (25.1, 38.3)
Symptoms	
- Shortness of breath	14 (77.8)
- Ankle swelling	8 (44.4)
- Fatigue	12 (66.7)
Heart failure history	
- Duration of HF < 5 years	7 (38.9)
- Time from feeling unwell to diagnosis median months (1st and 3rd quartiles)	7 (3, 12)
- Diagnosed in hospital	13 (72.2)

**Clinicians (n=27)**	
Women	
Men	21 (77.8)
Profession	6 (33.3
- Nurse/AHP	18 (66.7)
• Women	16 (88.8)
- Doctor	9 (33.3)
• Women	5 (55.6)
Time qualified > 10 years	25 (92.6
Work setting	
- HF community/outpatients	11 (40.7)
- HF hospital	8 (29.6)
- Other none HF setting or research	8 (29.6)
Experience in HF care (years)	
Between 1 and 3	4 (14.8)
Between 4 and 10	9 (33.3)
More than 10	14 (51.9)
Involved in HF diagnosis	17 (63.0)

Self-report information as part of 1 st survey. All data presented as n (%) unless otherwise stated. All data complete. Other none HF settings included acute or general cardiology, urgent care and general practice. Ethnicity: Patients and clinicians included individuals from non-White ethnic groups (South Asian, Black, and Mixed). Due to low numbers, specific counts are not reported in line with data governance requirements

### Delphi rounds

*missing data for survey responses can be found in supplementary file (S1 Table)

#### Round 1

In relation to delayed diagnosis, 11 sociodemographic factors achieved a mean score of > 3.0 (agree or strongly agree) (Table 3). The top 10 were carried forward to the second round. Factors such as older age, being male or affluent and ethnicity scored < 3.0 and were eliminated. Participants added two new factors: lack of access to appointments with a general practitioner or cardiologist and confusion about symptoms.

**Table 3:**
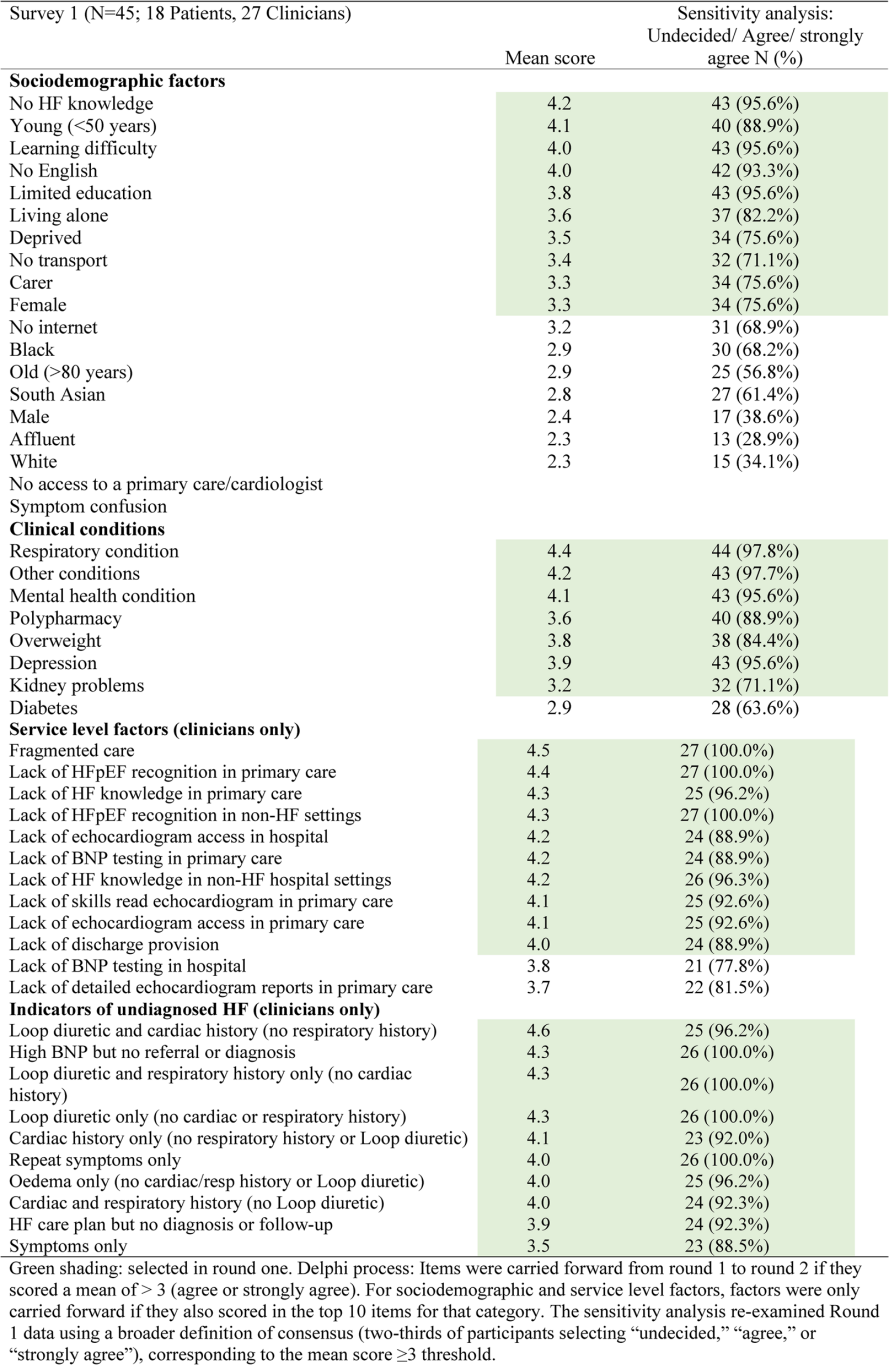
Consensus responses from survey 1

With the exception of diabetes, all clinical factors achieved a mean score of > 3.0 and were carried forward to Round 2. Similarly, all service-level factors scored > 3.0, but only the top 10 were carried forward. Among service-level factors, lack of NT-proBNP testing in hospital and lack of detailed echocardiogram reports in primary care scored the lowest and were excluded. Finally, all indicators of undiagnosed HF scored > 3.0 and were carried forward to Round 2.

Sensitivity analysis re-examining Round 1 data using a broader definition of consensus (two-thirds of participants selecting “undecided,” “agree,” or “strongly agree”), corresponding to the mean score ≥ 3 threshold, yielded the same set of retained items as the mean-based approach, indicating that findings were not sensitive to the choice of summary statistic (Table 3). Using only participants who completed all three rounds (*n* = 34) produced the same set of retained items as the full sample analysis (S2 Table). This indicates that attrition between rounds did not materially affect consensus stability or the final rankings.

#### Round 2

The second survey was returned by all 18 patients and 23 clinicians (Table 4).

**Table 4:**
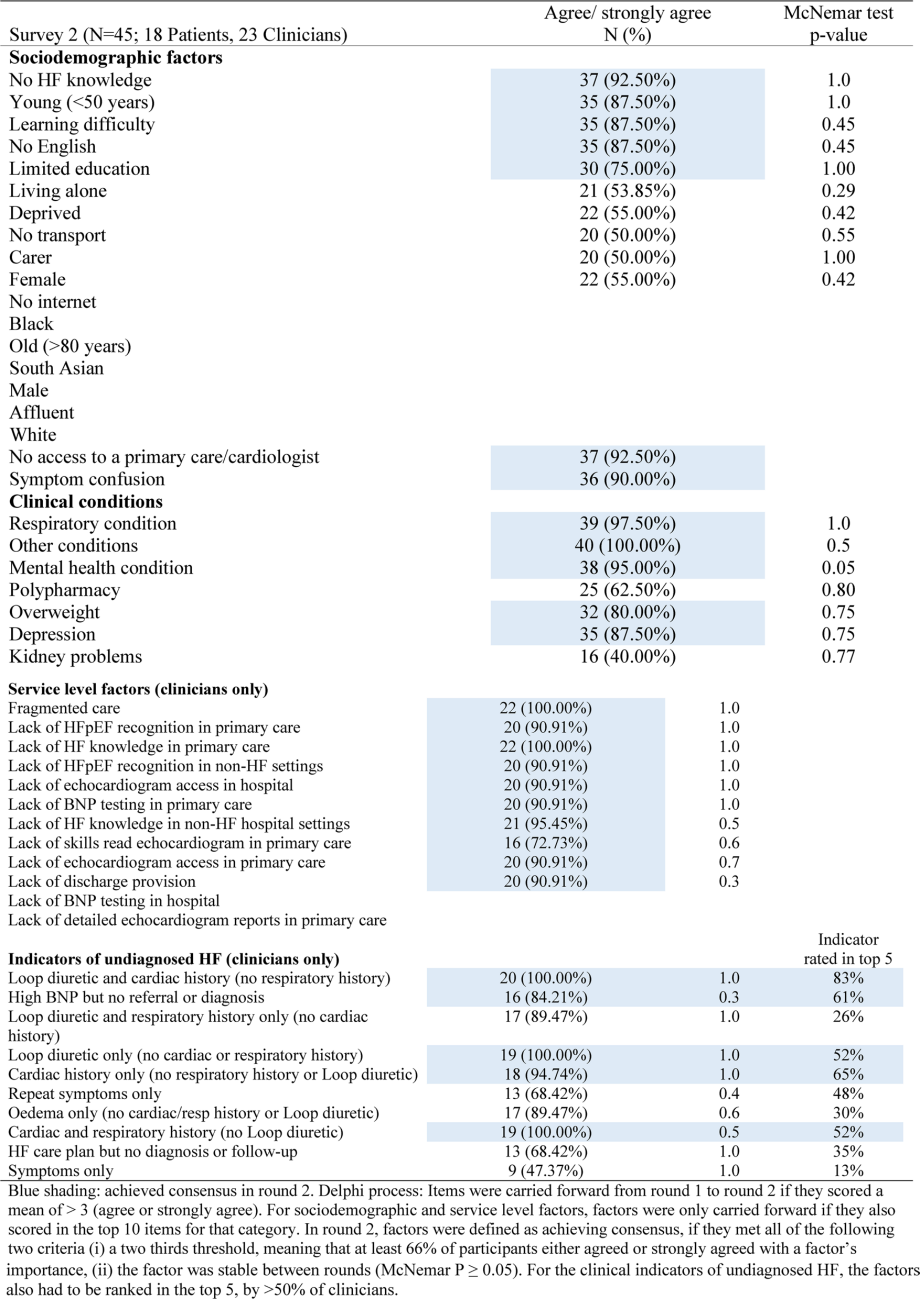
Consensus responses from survey 2

- Sociodemographic Factors: Five factors achieved the two-thirds threshold (66% agreement or strong agreement) and remained stable between rounds (*McNemar P ≥ 0.05*). Factors such as being deprived, female, a carer, living alone, or lacking transport did not meet the threshold and were excluded.
- Clinical Factors: Five factors met the two-thirds threshold and were stable between rounds. Polypharmacy and kidney problems did not meet the threshold and were eliminated.
- Service-Level Factors: All 10 service-level factors met the two-thirds threshold and were stable between rounds, so they were carried forward.
- Indicators of Undiagnosed HF: Most indicators met the two-thirds threshold and were stable, except for “symptoms only,” which was eliminated. Four additional indicators were removed because they were not ranked in the top 5 by > 50% of clinicians: *repeat symptoms only*,* loop diuretic and respiratory history only (no cardiac history)*,* oedema only (no cardiac/respiratory history or loop diuretic)*, and *HF care plan but no diagnosis or follow-up.*

#### Round 3

The third survey was returned by 17 patients and 17 clinicians (Table 5).

**Table 5:** Ranking in survey 3

Survey 3 (*N* = 34; 17 Patients, 17 Clinicians)	Ranking
Sociodemographic factors	
No HF knowledge	1
No access to primary care/cardiologist	2
Symptom confusion	3
Young < 50 years	4
Learning difficulty	5
No English language	7
Limited education	8
Clinical factors	
Other conditions	1
Respiratory condition	2
Mental health condition	3
Overweight	4
Depression	5
Service level factors (clinicians only)	
Lack of HF knowledge in primary care	1
Lack of BNP testing in primary care	2
Lack of HFpEF recognition in primary care	3
Fragmented care	4
Lack of echocardiogram access in primary care	5
Lack of HFpEF recognition in non-HF settings	6
Lack of echocardiogram access in hospital	7
Lack of HF knowledge in non-HF hospital settings	8
Lack of skills read echocardiogram in primary care	9
Lack of discharge provision	10
Indicators of undiagnosed HF (Clinicians only)	
High BNP but no referral or diagnosis	1
Loop diuretic and cardiac history (no respiratory history)	2
Loop diuretic only (no cardiac or respiratory history)	3
Cardiac history only (no respiratory history or Loop diuretic)	4
Cardiac and respiratory history (no Loop diuretic)	5

**Fig. 1:**
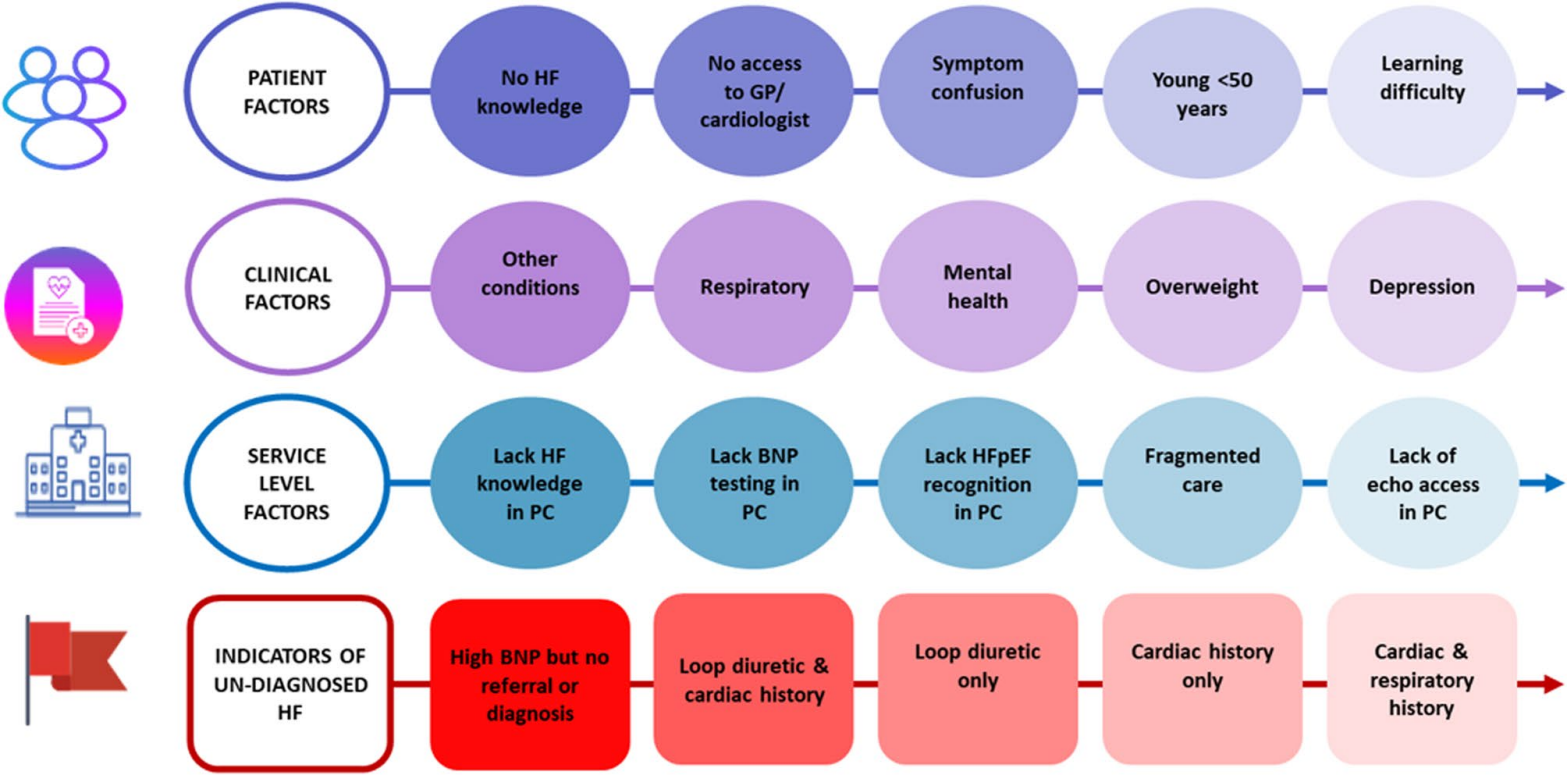
Top 5 ranked factors for delayed diagnosis. In the final Delphi round participants were asked to rank each set of factors in order of importance from 1 (most important) to the last item number (least important). The top 5 factors for each category are presented

In the final ranking (Fig. 1), the top five factors for delayed diagnosis were as follows:

- Sociodemographic factors:
  1. Patients lacking heart failure knowledge.
  2. No access to a general practitioner or cardiologist.
  3. Symptom confusion.
  4. Being young (< 50 years).
  5. Having a learning difficulty.
- Clinical Factors:
  1. Having other conditions.
  2. Having a respiratory condition.
  3. Having a mental health condition.
  4. Being overweight.
  5. Being depressed.
- Service-Level Factors:
  1. Lack of heart failure knowledge in general practice.
  2. Lack of NT-proBNP testing in general practice.
  3. Lack of Heart failure with preserved ejection fraction (HFpEF) recognition in general practice.
  4. Fragmented care.
  5. Lack of echo access in general practice.
- In relation to delayed diagnosis,
  1. High NT-proBNP but no referral or diagnosis.
  2. Loop diuretic and cardiac history (no respiratory history).
  3. Loop diuretic only (no cardiac or respiratory history).
  4. Cardiac history only (no respiratory history or loop diuretic).
  5. Cardiac and respiratory history (no loop diuretic).

## Discussion

Using a rigorous approach following COMET methods for COS development [25], this study has established a core set of sociodemographic, clinical and service level factors related to delayed diagnosis of heart failure (HF), alongside a priority list of five indicators of undiagnosed HF in primary care. These findings directly address international priorities to improve HF outcomes by identifying barriers to early diagnosis and opportunities for intervention.

Sociodemographic factors mainly reflected health literacy, with patients often lacking awareness of HF symptoms or confusing them with ageing or comorbidities. This aligns with evidence showing low global awareness of HF symptoms, with as few as 6% of the general public being aware [26], and with two-thirds of people failing to recognise their seriousness [27]. Such gaps in knowledge can delay health-seeking and are exacerbated by barriers in accessing timely GP and specialist appointments [28]. Most symptom awareness interventions focus on patients who have already developed HF [29], rather than recognising symptoms in the general population, highlighting the need for public education campaigns [30]. 

Clinical factors included multimorbidity and psychological comorbidities, which can complicate diagnosis and reduce health-seeking behaviour [21]. Previous research confirms that many patients presenting with breathlessness in primary care have unrecognised HF, particularly HFpEF [31, 32], suggesting the need for better screening of high-risk patients. Service-level barriers included limited knowledge and variable confidence among non-specialist clinicians, especially regarding HFpEF, and restricted access to diagnostic tests in primary care. In prior work [33], primary care clinicians were generally less confident than specialists in identifying various aspects of HF diagnosis, with both groups reporting lower confidence in diagnosing HFpEF. Additionally, approximately one-third of primary care clinicians did not consider echocardiography or laboratory tests essential for diagnosis or relevant to their role. In another study [21], general practitioners and nurses reported that they did not routinely diagnose HFpEF, relying instead on electrocardiograms and chest X-rays rather than guideline-recommended pathways. Limited access to NT-proBNP testing was reported by up to half of the clinician groups surveyed. Although accessibility to echocardiography was generally high, a lack of knowledge in interpreting results was reported to impede its utilisation. The prioritised indicators of undiagnosed HF, such as elevated NT-proBNP without follow-up, loop diuretic use, and cardiac history, highlight high-risk groups who warrant closer scrutiny. For example, patients prescribed loop diuretics without a confirmed HF diagnosis have worse outcomes [34, 35], underscoring the need for systematic review and timely referral in such cases.

Together, these findings highlight the need for multifaceted strategies: raising public awareness, enhancing clinician training (particularly in HFpEF), and implementing decision-support tools to improve recognition and referral. The World Health Organisation has emphasised the potential of e-health technologies to prevent health problems, improve care quality, reduce costs, and increase equitable access to healthcare [36]. The growing integration of technology in healthcare, including clinical decision support tools [37], offers an opportunity to address gaps in HF diagnosis. Such tools can assist clinicians by identifying patients at higher risk, providing diagnostic prompts, and guiding appropriate follow-up actions.

This study employed robust consensus methods to achieve agreement among HF patients and clinicians on a core set of patient, clinical, and service-level risk factors for delayed HF diagnosis, as well as a priority list of indicators of undiagnosed HF. It directly addresses international priorities to improve HF outcomes by identifying barriers to early diagnosis and appropriate testing. However, several limitations should be acknowledged. While participants were recruited from diverse settings, most were from White ethnic groups, and non-English-speaking participants were not included, potentially overlooking barriers unique to these populations. This limits generalisability to more ethnically and linguistically diverse populations, where cultural factors, communication barriers, and differential access to care may influence both symptom recognition and diagnostic pathways. Patient participants were also younger than typically observed in HF cohorts, with a higher proportion of women, likely reflecting the demographics of respondents from the national patient network. This may limit generalisability to older primary care populations, though all patients were under specialist care and able to draw on lived experience of delays in diagnosis. Among clinicians, representation was drawn from both primary and secondary care, but the number of medical respondents was lower than that of nursing respondents, which may have influenced factor prioritisation. While this reflects the multidisciplinary nature of HF care, it may underrepresent the perspectives of doctors, particularly those working in primary care. While purposive sampling of experienced clinicians may introduce some selection bias, this is an inherent and necessary feature of Delphi studies designed to generate expert consensus. Patients recruited through the national network self-reported HF, which could lead to some misclassification bias; however, they were all under the care of HF specialists, ensuring that their accounts reflected confirmed disease experience. Attrition between Delphi rounds, particularly among clinicians, may have affected the stability of consensus rankings, although retention rates were within the range reported in comparable Delphi studies and sensitivity analyses suggested minimal impact on final results. Although we highlighted the top five ranked factors for clarity, the full ranked list is presented to allow readers to consider alternative thresholds (e.g., top 3 or top 10). As the highest-priority items were consistently endorsed across participants, the overall interpretation of the findings is unlikely to be sensitive to the specific cut-off applied. Some of the proposed indicators, such as loop diuretic use as a marker of undiagnosed HF, should be considered hypothesis-generating rather than definitive, and require external validation in in real-world clinical datasets to assess feasibility, accuracy, and impact on diagnostic timeliness, before being applied in practice. Finally, this study was situated in UK primary care, where access to diagnostic tests is relatively good, the feasibility and applicability of the identified indicators may be more limited in health systems with restricted diagnostic capacity.

This study provides a potential framework for identifying patients at high risk of delayed HF diagnosis by identifying critical risk factors and indicators of undiagnosed HF. Whilst more research is required to fully understand the barriers and solutions for timely HF diagnosis, enhancing public awareness, increasing clinician knowledge, and adopting decision-support tools in primary care could significantly improve early HF detection and treatment, ultimately improving patient outcomes and reducing healthcare burdens.

## Supplementary Information

Supplementary Material 1.

## Data Availability

The datasets used and/or analysed during the current study are available from the corresponding author on reasonable request.
