# Supplementary material for: Developing core indicators for identifying people at risk of delayed heart failure diagnosis
Source: BMC Prim Care. 2025 Oct 16;26:316. doi: 10.1186/s12875-025-03024-4 (PMC12533351; doi:10.1186/s12875-025-03024-4)
Supplement: Supplementary file 1 — Supplementary Material 1. [file 12875_2025_3024_MOESM1_ESM.pdf]

Developing core indicators for identifying people at risk of delayed heart failure diagnosis.

Barber K, Bernhardt L, McCann G, Squire I, Miller C, Deaton C, Khunti K, Lawson C

Supplementary information

Supplementary table..2

S1 Table: Missing data in survey responses..2

S2 Table: Consensus responses from survey 1; sensitivity analysis following removal of participants who dropped out in rounds 2 or 3..4

Supplementary Figures 7

Figure 1: Patient questionnaire...7

Figure 2: Clinician questionnaire..7

Supplementary table

S1 Table: Missing data in survey responses

All partcipants	Survey 1<br>N=45	Survey 2<br>N=41
	Missing data presented as n (%)	
**Sociodemographic factors**		
No HF knowledge	0	1 (2)
Young (<50 years)	0	1 (2)
Learning difficulty	0	1 (2)
No English	0	1 (2)
Limited education	0	1 (2)
Living alone	0	2 (5)
Deprived	0	1 (2)
No transport	0	1 (2)
Carer	0	1 (2)
Female	0	1 (2)
No internet	0	0
Black	1 (2)	0
Old (>80 years)	1 (2)	0
South Asian	1 (2)	0
Male	1 (2)	0
Affluent	0	0
White	1 (2)	0
No access to a PRIMARY CARE/cardiologist	0	1 (2)
Symptom confusion	0	1 (2)
**Clinical factors**		
Respiratory condition	0	1 (2)
Other conditions	1 (2)	1 (2)
Mental health condition	0	1 (2)
Polypharmacy	0	1 (2)
Overweight	0	1 (2)
Depression	0	1 (2)
Kidney problems	0	1 (2)
Diabetes	1 (2)	0
Fragmented care	0	0
**Clinicians only**	**Survey 1 (n=27)**	**Survey 2 (n=23)**
**Service level factors**		
Lack HFpEF recognition in general practice	0	1 (4)
Lack HF knowledge in PRIMARY CARE	1 (4)	1 (4)
Lack HFpEF recognition in non-HF settings	0	1 (4)
Lack echocardiogram access in hospital	0	1 (4)
Lack BNP testing in primary care	0	1 (4)
Lack HF knowledge in non-HF hospital settings	0	1 (4)
Lack skills read echocardiogram in primary care	0	1 (4)
Lack echocardiogram access in primary care	0	1 (4)
Lack of discharge provision	0	1 (4)
Lack BNP testing in hospital	0	0
Lack of detailed echocardiogram reports in primary care	0	0

**Indicators in undiagnosed HF in patients presenting with HF symptoms**		
Loop diuretic and cardiac history (no respiratory history)	1 (4)	3 (13)
High BNP but no referral or diagnosis	1 (4)	4 (17)
Loop diuretic and respiratory history only (no cardiac history)	1 (4)	4 (17)
Loop diuretic only (no cardiac or respiratory history)	1 (4)	4 (17)
Cardiac history only (no respiratory history or Loop diuretic)	2 (7)	4 (17)
Repeat symptoms only	1 (4)	4 (17)
Oedema only (no cardiac/resp history or Loop diuretic)	1 (4)	4 (17)
Cardiac and respiratory history (no Loop diuretic)	1 (4)	4 (17)
HF care plan but no diagnosis or follow-up	1 (4)	4 (17)
Symptoms only	1 (4)	4 (17)

S2 Table: Consensus responses from survey 1; sensitivity analysis following removal of participants who dropped out in rounds 2 or 3

	All participants		Following removal of participants who dropped out in rounds 2 or 3	
	Survey 1 (N=45; 18 Patients, 27 Clinicians)		Survey 1 (N=34; 17 Patients, 17 Clinicians)	
	Mean score	Undecided/ Agree/ strongly agree N (%)	Mean score	Undecided/ Agree/ strongly agree N (%)
**Sociodemographic factors**			**Total**	
No HF knowledge	4.2	43 (95.6%)	4.2	32 (94.1%)
Young (<50 years)	4.1	40 (88.9%)	4.1	31 (91.2%)
Learning difficulty	4.0	43 (95.6%)	4.0	32 (94.1%)
No English	4.0	42 (93.3%)	3.9	31 (91.2%)
Limited education	3.8	43 (95.6%)	3.8	32 (94.1%)
Living alone	3.6	37 (82.2%)	3.6	28 (82.4%)
Deprived	3.5	34 (75.6%)	3.5	25 (73.5%)
No transport	3.4	32 (71.1%)	3.2	22 (64.7%)
Carer	3.3	34 (75.6%)	3.2	24 (70.6%)
Female	3.3	34 (75.6%)	3.2	25 (73.5%)
No internet	3.2	31 (68.9%)	3.0	21 (61.8%)
Black	2.9	30 (68.2%)	2.8	22 (66.7%)
Old (>80 years)	2.9	25 (56.8%)	3.1	21 (63.6%)
South Asian	2.8	27 (61.4%)	2.8	20 (60.6%)
Male	2.4	17 (38.6%)	2.4	14 (42.4%)
Affluent	2.3	13 (28.9%)	2.3	9 (26.5%)
White	2.3	15 (34.1%)	2.5	14 (42.4%)
No access to a primary care/cardiologist				
Symptom confusion				
**Clinical conditions**				
Respiratory condition	4.4	44 (97.8%)	4.4	33 (97.1%)
Other conditions	4.2	43 (97.7%)	4.2	32 (97.0%)

Mental health condition	4.1	43 (95.6%)	4.1	32 (94.1%)
Polypharmacy	3.6	40 (88.9%)	3.5	30 (88.2%)
Overweight	3.8	38 (84.4%)	3.7	28 (82.4%)
Depression	3.9	43 (95.6%)	3.9	32 (94.1%)
Kidney problems	3.2	32 (71.1%)	3.1	23 (67.6%)
Diabetes	3.0	28 (63.6%)	3.1	21 (61.8%)
**Service level factors (clinicians only)**				
Fragmented care	4.5	27 (100.0%)	4.5	17 (100.0%)
Lack of HFpEF recognition in primary care	4.4	27 (100.0%)	4.4	17 (100.0%)
Lack of HF knowledge in primary care	4.3	25 (96.2%)	4.4	17 (100.0%)
Lack of HFpEF recognition in non-HF settings	4.3	27 (100.0%)	4.3	17 (100.0%)
Lack of echocardiogram access in hospital	4.2	24 (88.9%)	4.1	14 (82.4%)
Lack of BNP testing in primary care	4.2	24 (88.9%)	4.0	14 (82.4%)
Lack of HF knowledge in non-HF hospital settings	4.2	26 (96.3%)	4.1	16 (94.1%)
Lack of skills read echocardiogram in primary care	4.1	25 (92.6%)	4.0	15 (88.2%)
Lack of echocardiogram access in primary care	4.1	25 (92.6%)	4.2	16 (94.1%)
Lack of discharge provision	4.0	24 (88.9%)	3.9	14 (82.4%)
Lack of BNP testing in hospital	3.8	21 (77.8%)	3.8	13 (76.5%)
Lack of detailed echocardiogram reports in primary care	3.7	22 (81.5%)	3.6	13 (76.5%)
**Indicators of undiagnosed HF (clinicians only)**				
Loop diuretic and cardiac history (no respiratory history)	4.6	25 (96.2%)	4.4	15 (93.8%)
High BNP but no referral or diagnosis	4.3	26 (100.0%)	4.2	16 (100.0%)
Loop diuretic and respiratory history only (no cardiac history)	4.3	26 (100.0%)	4.4	16 (100.0%)
Loop diuretic only (no cardiac or respiratory history)	4.3	26 (100.0%)	4.4	16 (100.0%)

Cardiac history only (no respiratory history or Loop diuretic)	4.1	23 (92.0%)	4.3	14 (93.3%)
Repeat symptoms only	4.0	26 (100.0%)	4.1	16 (100.0%)
Oedema only (no cardiac/resp history or Loop diuretic)	4.0	25 (96.2%)	4.2	16 (100.0%)
Cardiac and respiratory history (no Loop diuretic)	4.0	24 (92.3%)	4.2	16 (100.0%)
HF care plan but no diagnosis or follow-up	3.9	24 (92.3%)	3.9	15 (93.8%)
Symptoms only	3.5	23 (88.5%)	3.7	14 (87.5%)

Green shading: selected in round one. Delphi process: Items were carried forward from round 1 to round 2 if they scored a mean of > 3 (agree or strongly agree). For sociodemographic and service level factors, factors were only carried forward if they also scored in the top 10 items for that category.

Supplementary Figures

Figure 1: Patient questionnaire

Figure 2: Clinician questionnaire

Patient Heart Failure Diagnosis Consensus Survey: Round 1

You will be presented with a list of patient and clinical factors that may delay diagnosis of heart failure.

Please rate each factor between 1 and 5, according to the questions asked.

Patient Heart Failure Diagnosis Consensus Survey: Round 1

1. The following **patient factors** are **important** factors that might delay diagnosis of heart failure.

	1. STRONGLY DISAGREE	2. DISAGREE	3. UNDECIDED	4.AGREE	5. STRONGLY AGREE
Being female	◯	◯	◯	◯	◯
Being male	◯	◯	◯	◯	◯
Being old (above 80 years)	◯	◯	◯	◯	◯
Being young (below 50 years)	◯	◯	◯	◯	◯
Being White	◯	◯	◯	◯	◯
Being South Asian	◯	◯	◯	◯	◯
Being Black	◯	◯	◯	◯	◯
Being from an affluent (well off) background	◯	◯	◯	◯	◯
Being from a poor	◯	◯	◯	◯	◯

background					
Living alone	○	○	○	○	○
Being a carer for someone else	○	○	○	○	○
Having no transport	○	○	○	○	○
Not having access to the internet	○	○	○	○	○
Having learning difficulties	○	○	○	○	○
Not speaking English	○	○	○	○	○
Having limited education	○	○	○	○	○
Not knowing about heart failure/ heart failure symptoms	○	○	○	○	○

2. Are there any important patient factors that are missing? Please add one per box.

Factor 1 [ ]

Factor 2 [ ]

Factor 3 [ ]

Factor 4 [ ]

3. The following **clinical factors** are **important** factors that might delay diagnosis of heart failure.

	1. STRONGLY DISAGREE	2. DISAGREE	3. UNDECIDED	4.AGREE	5. STRONGLY AGREE
Having other conditions	○	○	○	○	○
Having a respiratory condition	○	○	○	○	○
Having a mental health condition	○	○	○	○	○
Being depressed	○	○	○	○	○
Having diabetes	○	○	○	○	○
Having kidney problems	○	○	○	○	○
Being on lots of tablets	○	○	○	○	○
Being overweight	○	○	○	○	○

4. Are there any important clinical factors that are missing? Please add one per box.

Factor 1 [ ]

Factor 2 [ ]

Factor 3 [ ]

Factor 4 [ ]

Patient Heart Failure Diagnosis Consensus Survey: Round 1

Thank you for completing our survey!

There are two further surveys in this study.

The next one will be sent to you next month. It will show you the results from the whole group (patients and clinical staff) and ask you to score your responses again.

* 5. Please indicate below whether you are willing to continue to participate in this study

○ Yes, I am happy to continue to participate in the study

○ No, please don't send me any further surveys

6. If you have agreed to continue in our study, please indicate whether you are still happy to receive reminders before and after the survey submission date.

○ Yes, I am happy to receive reminders

○ No, please don't send me reminders

Clinician Heart Failure Diagnosis Consensus Survey: Round 1

SECTION 1

You will be presented with a list of sociodemographic, clinical and service level factors that may delay diagnosis of heart failure.

Please rate each factor between 1 and 5, according to the questions asked.

Clinician Heart Failure Diagnosis Consensus Survey: Round 1

1. The following are **important sociodemographic** factors that might delay diagnosis of heart failure.

	1. STRONGLY DISAGREE	2. DISAGREE	3. UNDECIDED	4.AGREE	5. STRONGLY AGREE
Being female	◯	◯	◯	◯	◯
Being male	◯	◯	◯	◯	◯
Being old (above 80 years)	◯	◯	◯	◯	◯
Being young (below 50 years)	◯	◯	◯	◯	◯
Being White	◯	◯	◯	◯	◯
Being South Asian	◯	◯	◯	◯	◯
Being Black	◯	◯	◯	◯	◯
Being from an affluent background	◯	◯	◯	◯	◯
Being from a deprived	◯	◯	◯	◯	◯

background					
Living alone	◯	◯	◯	◯	◯
Being a carer for someone else	◯	◯	◯	◯	◯
Having no transport	◯	◯	◯	◯	◯
Not having access to the internet	◯	◯	◯	◯	◯
Having learning difficulties	◯	◯	◯	◯	◯
Not speaking English	◯	◯	◯	◯	◯
Having limited education	◯	◯	◯	◯	◯
Not knowing about heart failure/ heart failure symptoms	◯	◯	◯	◯	◯

2. Are there important sociodemographic factors that are missing? Please add one per box

Factor 1 [ ]

Factor 2 [ ]

Factor 3 [ ]

Factor 4 [ ]

3. The following are **important clinical factors** that might delay diagnosis of heart failure.

	1. STRONGLY DISAGREE	2. DISAGREE	3. UNDECIDED	4.AGREE	5. STRONGLY AGREE
Having other conditions	○	○	○	○	○
Having a respiratory condition	○	○	○	○	○
Having a mental health condition	○	○	○	○	○
Being depressed	○	○	○	○	○
Having diabetes	○	○	○	○	○
Having kidney problems	○	○	○	○	○
Being on lots of tablets	○	○	○	○	○
Being overweight	○	○	○	○	○

4. Are there important clinical factors that are missing? Please add one per box

Factor 1 [ ]

Factor 2 [ ]

Factor 3 [ ]

Factor 4 [ ]

5. The following are **important service level factors** that might delay diagnosis of heart failure.

	1. STRONGLY DISAGREE	2. DISAGREE	3. UNDECIDED	4.AGREE	5. STRONGLY AGREE
Lack of access to BNP in general practice	○	○	○	○	○
Lack of					

access to BNP in hospital	◯	◯	◯	◯	◯
Lack of access to echoe in general practice	◯	◯	◯	◯	◯
Lack of access to echoe in hospital	◯	◯	◯	◯	◯
Lack of detail in echoe reports to general practice	◯	◯	◯	◯	◯
Lack of the required skills to interpret echoe reports in general practice	◯	◯	◯	◯	◯
Lack of heart failure knowledge in general practice	◯	◯	◯	◯	◯
Lack of heart failure knowledge in non-HF hospital settings	◯	◯	◯	◯	◯
Fragmented care (multiple specialists/ departments)	◯	◯	◯	◯	◯
Lack of HFpEF recognition in general	◯	◯	◯	◯	◯

practice					
Lack of HFpEF recognition in non-HF hospital settings	○	○	○	○	○
Inadequate discharge provision/ care plan in hospital	○	○	○	○	○

6. Are there important service level factors that are missing? Please add one per box

Factor 1	
Factor 2	
Factor 3	
Factor 4	

Clinician Heart Failure Diagnosis Consensus Survey: Round 1

SECTION 2

You will now be presented with a list of indicators for undiagnosed heart failure in general practice patients.

Please rate each factor between 1 and 5, according to the questions asked.

Clinician Heart Failure Diagnosis Consensus Survey: Round 1

7. In patients in general practice without a diagnosis of heart failure currently, the following are **important indicators** of undiagnosed heart failure.

	1. STRONGLY DISAGREE	2. DISAGREE	3. UNDECIDED	4.AGREE	5. STRONGLY AGREE
With HF symptoms (dyspnoea, ankle swelling, fatigue) **and** loop diuretic **and** history of IHD/AF	○	○	○	○	○
With HF symptoms **and** loop diuretic, **without** IHD/AF or respiratory disease (COPD/Asthma/ILD)	○	○	○	○	○
With HF symptoms **and** loop diuretic **and** history of respiratory disease but **without** IHD/AF	○	○	○	○	○
With HF symptoms **and** history of IHD/AF but **without** respiratory disease or loop diuretic	○	○	○	○	○
With HF symptoms **and** history of IHD/AF **and** respiratory disease but **without** a Loop diuretic.	○	○	○	○	○
With symptoms **and** oedema but **without** IHD/ AF/ respiratory disease/ loop diuretic	○	○	○	○	○
With raised NT-proBNP but **without** referral or HF diagnosis outcome	○	○	○	○	○
With HF clinic referral/ possession of care plan/ named HF contact, but **without** outcome or follow-up.	○	○	○	○	○
With first presentation of HF symptoms ONLY (**without** oedema/IHD/AF/Respiratory disease or Loop diuretic).	○	○	○	○	○
With repeat presentation of HF symptoms ONLY (**without** oedema/IHD/AF/Respiratory disease or Loop diuretic).	○	○	○	○	○

8. Are there important indicators that are missing? Please add one per box

Factor 1	
Factor 2	
Factor 3	
Factor 4	

Clinician Heart Failure Diagnosis Consensus Survey: Round 1

Thank you for completing our survey!

There are two further surveys in this study.

The next one will be sent to you next month. It will show you the average section 2 and 3 results from all the participants and ask you to score your responses again.

* 9. Please indicate below whether you are willing to continue to participate in this study

◯ Yes, I am happy to continue to participate in the study

◯ No, please don't send me any further surveys

10. If you have agreed to continue in our study, please indicate whether you are still happy to receive reminders before and after the survey submission date.

◯ Yes, I am happy to receive reminders

◯ No, please don't send me reminders
